# Skin Metastases As First Manifestations of Adenocarcinoma of the Lung: A Case Report

**DOI:** 10.7759/cureus.24834

**Published:** 2022-05-08

**Authors:** Rafael Everton Assunção Ribeiro da Costa, Erlan Clayton Xavier Cavalcante, Maria Clara Amorim Silva, Augusto Cesar Maia Rio Lima Silveira, Lina Gomes dos Santos, Ary Oliveira Pires, Sabas Carlos Vieira

**Affiliations:** 1 Health Science Center, State University of Piauí, Teresina, BRA; 2 Medical School, UNINOVAFAPI University Centre, Teresina, BRA; 3 Pathology, San Marcos Hospital, Teresina, BRA; 4 Radiology, UDI 24-hour Clinic, Teresina, BRA; 5 Tocogynecology, Oncocenter, Teresina, BRA

**Keywords:** case reports, adenocarcinoma of lung, lung cancer, skin, metastasis

## Abstract

Lung cancer is the second most common malignancy worldwide, accounting for the highest number of cancer deaths. Advanced lung cancer may infrequently appear as skin metastasis and this may be the first sign of the disease. In these cases, survival is low and prognosis is poor. The aim of this study is to report a case of adenocarcinoma of the lung where the earliest manifestations were skin metastases to the face, cervical region, and chest. A 67-year-old male, former smoker, and alcoholic was referred to the oncology center for investigation of a primary tumor site, presenting with skin lesions suggestive of metastasis to the face, cervical region, and chest. Computed tomography (CT) scan of the chest, cholangioresonance, breast ultrasonography, colonoscopy, upper GI endoscopy, and magnetic resonance imaging of the brain were performed. Imaging studies revealed disseminated cancer with a potential primary site in the right lung. Positron emission tomography (PET)-CT scan demonstrated secondary implants and was consistent with primary right lung cancer. The patient underwent a right lung biopsy of the skin and breast and axillary lymph nodes. A solid subtype of adenocarcinoma with metastases to the skin and axillary nodes was confirmed. Due to widespread metastatic disease, the case was conducted using strategies including chemotherapy and palliative radiotherapy for symptomatic control. At about 6 months of follow-up care, the patient died. In the elderly, periodical cancer screening is important, especially in patients with major risk factors (e.g., history of smoking). Some cancers may be virtually silent and manifest themselves only at advanced stages beyond treatment possibilities.

## Introduction

Global Cancer Statistics 2020 estimated that lung cancer was the second most common cancer worldwide in 2020, excluding nonmelanoma skin cancer, and represented 11.4% of all cases. Furthermore, lung cancer was responsible for the highest number of cancer deaths in that same year, with a total of 18% of cancer deaths [1].

Lung cancer has a very poor prognosis and the 5-year overall survival rate is around 15%. The most common histologic subtypes are: adenocarcinoma, squamous cell carcinoma, small cell carcinoma, and large cell carcinoma. The most frequent metastatic sites are the brain, vertebral column, bones, liver, and adrenal glands [2,3].

Although an uncommon phenomenon, advanced lung cancer may manifest itself with skin metastases in 1-12% of patients. In very rare cases, skin metastases may be the earliest manifestations. In these cases, the prognosis is quite poor, the mean survival rate is only 3-5 months, and patients are usually preterminal. Skin metastases due to lung cancer are mainly located in the chest, abdomen, head, and neck [4].

The aim of this study is to report a case of adenocarcinoma of the lung in which the earliest manifestations were skin metastases to the face, cervical region, and chest. We present the following case in accordance with the CAse REport guidelines (CARE) reporting checklist.

## Case presentation

**Figure 1:**
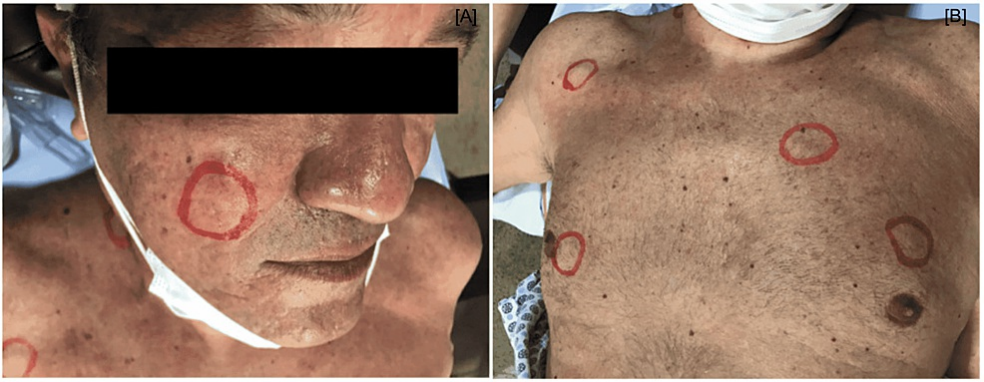
Skin lesions on the face, cervical region (A), and chest (B) presented by the patient.

A 67-year-old male former smoker (40 pack-years; quit smoking 20 years ago), alcoholic for 50 years, was referred to the oncology center in October 2021 for diagnostic investigation of the primary tumor site, since he had skin lesions suggestive of metastasis to the face, cervical region (Figure 1A), and chest (Figure 1B). The patient had no complaints and was in good physical condition. He had a history of systemic arterial hypertension (SAH) and type 2 diabetes mellitus (DM2) under treatment for 10 years. Family history of cancer in first-degree relatives was negative. CT scan of the chest revealed a solid mass in the upper lobe of the right lung, measuring 2.1 cm with irregular margins, along with other scattered solid pulmonary micronodules, measuring up to 0.2 cm and lymphadenopathy in mediastinal, pre-hilar, and paratracheal chains, with a necrotic center, consistent with secondary disease.

Cholangioresonance showed nodules in hepatic segment VIII, adrenal glands and greater omentum, and bone lesions in the lumbar vertebral spinal column, all suggestive of secondary disease. Breast ultrasonography (USG) showed solid breast masses and axillary lymph node involvement (nodule of 1 × 0.5 × 0.9 cm in the right breast and a nodule of 0.9 × 0.3 × 0.5 cm in the left breast; two atypical right axillary lymph nodes, measuring 1 × 0.9 cm and 0.9 × 0.7 cm, and left node measuring 1 × 0.8 cm). Upper GI endoscopy (UGIE) and magnetic resonance imaging (MRI) of the brain were normal.

**Figure 2:**
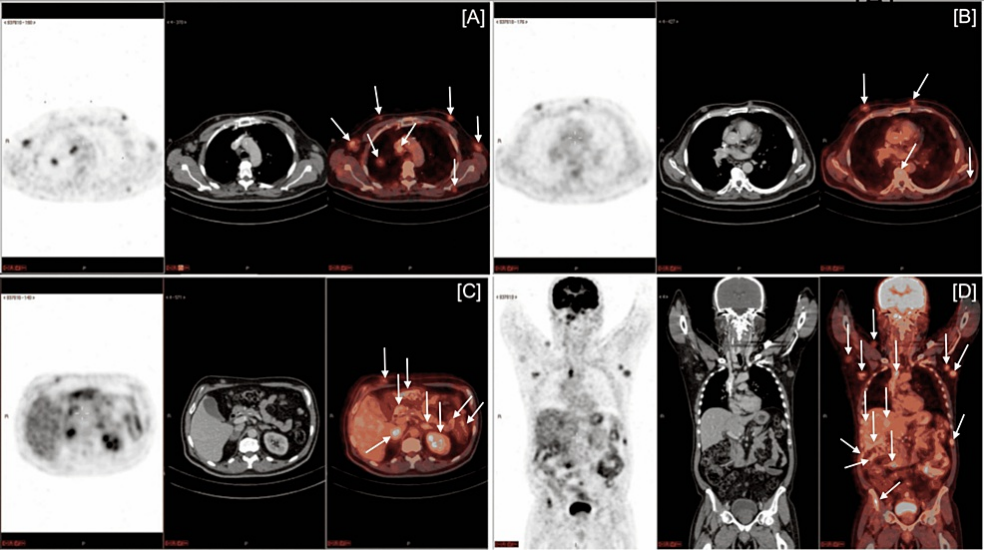
Positron emission computed tomography (PET-CT) scan images. Explanatory Note: Arrows indicate the most prominent uptake areas on PET-CT.

In view of the evidence of disseminated cancer suggesting that the potential primary site was the right lung, positron emission tomography (PET-CT) scan was performed (Figures 2A-2D), revealing an increased metabolic expression of the spiculated lung nodule in the right upper lobe, measuring about 2 cm (standard uptake value (SUV): 3.3), nodal lesions in the right pulmonary hilum, and mediastinum bilaterally, measuring up to 3.4 cm (maximum SUV: 5.1), bilateral adrenal lesions, measuring up to 3.6 cm at the right (maximum SUV: 4), hepatic nodule in segment VIII, measuring 2.4 cm (maximum SUV: 3.2), sparse solid peritoneal, retroperitoneal and extraperitoneal nodules, measuring up to 1.5 cm (SUV: 2), sparse osteolytic lesion in axial and appendicular skeleton, highlighting the vertebral column, left clavicle, sternum, iliac, and femurs (SUV: 5.2), sparse subcutaneous and muscular lesions, highlighting the pectoral/mammary, axillary, dorsal, abdominal, lumbar, and perineal regions, measuring up to 3 cm (maximum SUV: 3.7), and increased prostate elevating the pelvic floor, which showed thickened walls. PET-CT results were consistent with primary cancer of the right lung.

**Figure 3:**
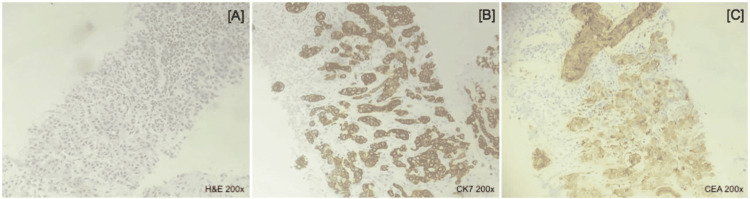
Histopathology and immunohistochemistry studies. (A): Hematoxylin-eosin (H&E); (B): cytokeratin 7 (CK7); (C): carcinoembryonic antigen (CEA). 200× magnification in all slides.

Histopathological exam of the right lung biopsy revealed a non-small cell lung carcinoma (Figure 3A). Immunohistochemistry (IHC) analysis of the lung parenchyma sample showed a tumor consisting of a proliferation of cuboidal/vacuolated cells, isolated and/or arranged in solid nests or outlining glandular architecture, permeated by areas of tumor necrosis. Tumor cells significantly expressed CK7 (Figure 3B) and carcinoembryonic antigen (CEA) (Figure 3C), also focally expressing, CA 19-9. The investigation was negative for the remaining markers tested (Glypican 3, Napsin A, thyroid transcription factor-1 (TTF-1)). Clinical and morphological findings indicated a solid subtype of lung adenocarcinoma. Biopsy of the skin, breast masses, and axillary lymph nodes also showed a metastatic tumor of pulmonary origin.

The first chemotherapy cycle with cisplatin associated with pemetrexed was administered on January 7, 2022. The second cycle was administered on February 28, 2022. Due to pain in the upper part of the pelvis, palliative radiotherapy with 30 Gy in 10 fractions was indicated for this region and it began after February 2022. The patient showed improvement in bone pain. After about 6 months of follow-up care (October 14, 2021-April 13, 2022) in the oncology unit, the patient died.

## Discussion

Around 80-90% of all skin metastases in adults originate from malignancies located in internal organs, highlighting lung cancer, breast cancer, melanocytes, oral cavity, colon, kidneys, ovaries, and stomach. In men, skin metastases derive mainly from lung cancer (12-28%), gastrointestinal cancer (11-19%), and melanoma (13-32%). In contrast, lung cancer in women represents the fifth most common site of skin metastases (4%), followed by breast cancer (69%), colon cancer (9%), melanoma (5%), and ovarian cancer (4%) [2]. Skin metastases derived from lung cancer are uncommon. Gogia et al.’s data from a meta-analysis of seven studies showed that the global incidence of skin metastases derived from lung cancer was 5.3%. In about 20-60% of these cases, skin metastases may occur before or during lung cancer diagnosis and is a warning sign that is particularly important in patients who are smokers or former smokers [5].

The most common sites for the occurrence of skin metastases derived from lung cancer are: chest (28.4%), abdomen (20.2%), limbs (12%), neck (11%), back (11%), scalp (7%), pelvis (6%), and face (5%) [6]. Morphological aspects can manifest as fibrotic processes, bullae, cellulitis-like lesions, zosteriform lesions, ulcerations, or nodules, which can be fixed or mobile. Lesions can be painless, presenting a color spectrum of color ranging from red to black, usually varying in size from 2 mm to 6 cm [2,7]. The main differential diagnoses of this clinical picture include: squamous cell carcinomas, basal cell and Merkel cell carcinomas, amelanotic melanomas, carcinoid tumors, and metastatic carcinomas of the lung [7].

Clinical suspicion of lung cancer may be challenging, since it may have a quiescent nature. Furthermore, some metastatic lesions may not strongly suggest a primary pulmonary site. In these types of presentations, diagnosis is usually late and occurs in the presence of diverse distant metastases [2,7]. For cases where it is possible to correlate the occurrence of certain metastatic lesions with a primary pulmonary site, treatment can be performed before the occurrence of widespread distant metastases [7]. In the case described in this study, skin metastases in a former smoker suggested the potential dissemination of a primary lung tumor, facilitating the performance of diagnostic investigation to determine a more appropriate treatment. Nevertheless, the cancer was already at a very late stage with widespread distant metastases, and the only treatment option available was the use of strategies to control symptoms, such as chemotherapy and palliative radiotherapy.

A definitive diagnosis of lung cancer is performed by histopathological and IHC analyses. In the sample of lung parenchyma removed during the biopsy of the patient in this case report, the histopathological exam was inconclusive as to the histological subtype, indicating a potential non-small cell carcinoma, that would be confirmed by IHC. IHC has a solid role in the definitive diagnosis of less differentiated lung tumors, using a series of markers to determine tumor origin, including CK7, CK20, TTF-1, and CDX-2, among others. In the present report, CK7 and CEA markers were positive, suggesting in conjunction with the histopathological exam that it was a solid subtype of lung adenocarcinoma. However, the most sensitive and specific marker in IHC for these cases is TTF-1, present in 60-75% of these tumors [5].

It is worth mentioning that the anatomy of the lung has few nerve endings. Tumor growth in this region occurs frequently unnoticed, until more evident symptoms or signs occur, including pain, hemoptysis, and obstructive pneumonia, among other systemic signs. The use of resources, such as CT scans, may contribute to the early detection of smaller and asymptomatic tumors, resulting in a more favorable prognosis [8]. In the case under discussion, the patient had no signs or symptoms that could indicate imaging studies before skin metastases appeared at a late stage of cancer dissemination. PET-CT detects increases in glucose metabolism in cancer cells, in comparison to normal cells, aiding in the diagnosis and evaluation of occult or known metastases. The high specificity and sensitivity of PET-CT scan also permits staging of lung cancer patients, providing better guidance to manage these cases [9]. In the patient of this case report, a PET-CT scan was important for clinical management, revealing various metastatic sites and pointing to the primary lung site.

Treatment of lung cancer with skin metastases may use several strategies, depending on the clinical, anatomical, and pathological characteristics of each tumor. It has been well-established that the appearance of only a single metastatic skin lesion demands surgical resection alone or in combination with radiotherapy and/or chemotherapy since there is evidence that surgical treatment is responsible for improved patient survival in these cases [2]. However, in cases of multiple skin metastases, chemotherapy is the primary treatment. The response of skin lesions serves as a marker of treatment response, despite the usually low survival in these cases [10]. Radiotherapy can also be used alone or in combination with chemotherapy in these cases. Nevertheless, radiotherapy is usually not effective and is palliative, as occurred in the case described in this study [11].

Patient prognosis with lung cancer and skin metastases is poor and survival is usually a few months only, ranging from 3 to 15 months, depending on the clinical presentation of each patient, as well as the therapeutic approach used. In some cases, palliative strategies help to prolong survival [2,4,5,7,10,11]. At about 6 months of follow-up care in the oncology unit, the patient analyzed in this study died.

## Conclusions

In this study, advanced adenocarcinoma of the lung, initially manifested as skin metastasis, shows the importance of cancer screening in elderly patients, particularly those with major risk factors, such as a history of smoking. Some cancers infrequently have a silent and less specific nature. Clinical manifestation of these tumors may occur only at late preterminal stages beyond any treatment possibility.
